# Fellow-eye asymmetry on optical coherence tomography angiography and thickness parameters in unilateral pseudoexfoliation syndrome

**DOI:** 10.5935/0004-2749.20220060

**Published:** 2025-08-21

**Authors:** Isil Pasaoglu, Zeynep Kayaarasi Ozturker, Selcen Celik, Bulut Ocak, Tekin Yasar

**Affiliations:** 1 Beyoglu Eye Training and Research Hospital, University of Health Sciences, Istanbul, Turkey; 2 Ophthalmology Department, Faculty of Medicine, Baskent University, Istanbul, Turkey

**Keywords:** Exfoliation syndrome, Glaucoma, Macular ganglion cell complex, Optical Coherence Tomography Angiography, Retinal vessel density, Síndrome de exfoliação, Glaucoma, Complexo de células ganglionares maculares, Angiofluoresceinografia, Tomografia de coerência óptica, Densidade de vasos retinianos

## Abstract

**Purpose:**

To investigate inter-eye retinal vessel density and thickness asymmetry in unilateral pseudoexfoliation syndrome and understand its use for the early detection of glaucoma.

**Methods:**

Thirty patients with unilateral pseudoexfoliation syndrome were enrolled in our study. Optical coherence tomography angiography macular scans were used measure the retinal vessel density, and optical coherence tomography scans were used to assess the thickness parameters of the peripapillary retinal nerve fiber layer and the macular ganglion cell complex. Inter-eye asymmetry was determined by taking the absolute value of the difference in the vessel density and thickness parameters between the pseudoexfoliation syndrome eye and fellow eye.

**Results:**

The mean patient age was 64.20 ± 7.05 y in the study group. Inter-eye asymmetry in the peripapillary retinal nerve fiber layer thickness and macular ganglion cell complex measurements were significant in the study group (p=0.03 and p=0.001, respectively). The vessel density of the macular superficial inner region was significantly lower in eyes with pseudoexfoliation syndrome than in fellow eyes (p=0.035). However, there was no inter-eye asymmetry in the central and full region macular superficial vessel density of eyes with pseudoexfoliation syndrome and fellow eyes (p>0.05).

**Conclusions:**

Retinal vessel density can be evaluated using optical coherence tomography angiography measurements. There was inter-eye asymmetry in the inner region macular superficial vessel density, peripapillary retinal nerve fiber layer, and macular ganglion cell complex thickness of the unilateral pseudoexfoliation syndrome eyes and fellow eyes. Further studies on a larger number of subjects might provide more clarity regarding the relationship between the inter-eye asymmetry of the retinal vessel density and thickness parameters with early detection of glaucomatous damage.

## Abstract (pt)

**Objetivo:**

Investigar a densidade dos vasos interoculares da retina e assimetria na espessura na síndrome de pseudoexfoliação unilateral e o seu uso para a detecção precoce de glaucoma.

**Métodos:**

Trinta pacientes com síndrome de pseudoexfoliação unilateral foram incluídos no estudo. As varreduras maculares de angiografia por tomografia de coerência óptica mediram a densidade dos vasos da retina, e as varreduras por tomografia de coerência óptica obtiveram parâmetros de espessura da camada de fibras nervosas da retina peripapilar e do complexo macular de célula ganglionar. A assimetria interocular foi determinada tomando o valor absoluto da diferença entre o olho da síndrome de pseudoexfoliação e o olho oposto nos parâmetros de densidade e espessura dos vasos.

**Resultados:**

A média de idade foi 64,20 ± 7,05 anos no grupo de estudo. A assimetria interocular na espessura da camada de fibra nervosa da retina peripapilar e as medidas do complexo macular de célula ganglionar foram estatisticamente significativas no grupo de estudo (p=0,03 e p=0,001, respectivamente). Para os olhos com síndrome de pseudoexfoliação, a densidade do vaso da região macular superficial interna foi significativamente menor do que em olhos opostos (p=0,035). No entanto, não houve assimetria interocular estatisticamente significativa na densidade macular dos vasos superficiais da região central e completa entre os olhos da síndrome de pseudoexfoliação e os olhos opostos (p>0,05).

**Conclusões:**

A densidade dos vasos da retina pode ser avaliada por medidas de angiografia por tomografia de coerência óptica. Houve assimetria interocular na densidade macular do vaso superficial da região interna, camada de fibra nervosa da retina peripapilar e espessura do complexo macular de célula ganglionar entre olhos com síndrome de pseudoexfoliação unilateral e olhos opostos. Novos estudos com um número maior de indivíduos podem fornecer a relação entre a assimetria interocular da densidade do vaso da retina e os parâmetros de espessura com detecção precoce de dano glaucomatoso.

## INTRODUCTION

Pseudoexfoliation (PEX) syndrome is a basement membrane disorder that occurs with age and is characterized by excessive microfibril deposition and abnormal elastosis in various regions of the eye, such as the conjunctiva, corneal endothelium, iris, anterior lens capsule, zonules, ciliary body, and trabecular meshwork^(1)^.

The accumulation of pseudoexfoliative material and deposits of released pigments in the anterior chamber have been associated with injury to the trabecular meshwork endothelium that leads to disruption of aqueous humor circulation and development of PEX glaucoma^(2)^.

It is known that damage due to glaucoma is more common in eyes with pseudoexfoliative material than that in those without pseudoexfoliative material^(3)^. In the Early Manifest Glaucoma Trial, PEX material was considered the most important independent risk factor for glaucoma progression^(4)^. While glaucoma is usually a bilateral disease, it is generally asymmetric in the initial stages^(5)^ and is defined as an early sign of glaucomatous damage^(6)^. Initially, PEX material accumulation and glaucomatous changes may occur asymmetrically and unilaterally in PEX syndrome. However, 15%-40% of patients have bilateral involvement within 5-10 y^(7)^. Glaucoma can be asymptomatic until the advanced stages of the disease and cause irreversible blindness^(8)^. The visual field may be preserved until most of the retinal ganglion cells (RGCs) are damaged^(9)^. Therefore, being aware of the early changes during the disease is crucial for the early detection of glaucomatous damage. RGCs mostly exist in the macula; therefore, methods that can measure glaucomatous changes associated with RGC loss enable early and accurate recognition of glaucoma^(10)^.

Optical coherence tomography angiography (OCT-A) is a non-invasive procedure that allows the evaluation of retinal microvasculature. Recent studies that have used OCT-A have found decreased vascularity in the peripapillary region in glaucoma^(11,12)^. In addition, retinal vascular density was reportedly highest in healthy eyes, followed by that in eyes with suspected glaucoma and in eyes with mild primary open-angle glaucoma^(13)^. These results suggest that retinal vascular dropout may occur earlier in glaucomatous eyes. To our knowledge, inter-eye asymmetry of retinal vessel density and macular ganglion cell complex (mGCC) have not been studied previously in eyes with unilateral PEX syndrome.

In the present study, we performed inter-eye investigation and measured the macular superficial vessel density and peripapillary retinal nerve fiber layer (RNFL) thickness. Further, we assessed mGCC measurement asymmetry using optical coherence tomography (OCT). The OCT-A system was used to assess healthy subjects with unilateral PEX syndrome and its use for the early detection of glaucoma.

## METHODS

This cross-sectional study was performed as per the tenets of the Declaration of Helsinki and was approved by the local ethics committee. Informed consent was obtained from all the enrolled patients.

All the patients were subjected to a complete ophthalmologic examination, including the best-corrected visual acuity (BCVA), a refraction test, slit-lamp biomicroscopy, Goldmann applanation tonometry, gonioscopy, dilated optic disc, and fundus examination. Central corneal thickness was measured using ultrasonic pachymetry (DGH-550, DGH Technology Inc., Exton, PA, USA), and 30-2 program visual field testing was performed using the Humphrey perimetry (HFATM II; Humphrey Instruments Inc., San Leandro, CA, USA).

The study included patients who attended the out patient clinic of a tertiary eye hospital and had findings of unilateral PEX syndrome. The presence of clinical PEX syndrome was defined as having pseudoexfoliative material deposits on the edge of the pupil and/or the lens capsule, intraocular pressure (IOP) of less than 21 mmHg with no history of IOP increase, open iridocorneal angles upon gonioscopy, BCVA of 20/40 or better, a normal appearance of the optic nerve head, and normal visual field test results.

Patients with chronic systemic diseases, such as diabetes mellitus and arterial hypertension; a BCVA <20/40; refraction of > ± 5.0 diopters sphere and ± 3.0 diopters cylinder; history of ocular trauma or surgery; an inflammatory eye disease; or retinal diseases, such as diabetic retinopathy and macular degeneration, were excluded.

### Optical coherence tomography

OCT (Cirrus HD-OCT, Carl Zeiss Meditec, Inc., Dublin, CA, USA) was used to perform the peripapillary RNFL measurements along a 3.4-mm diameter circle centered on the optic disc. Images with non-centered scans and inaccurate segmentation of the RNFL that could not be manually corrected were excluded from the analyses.

Ganglion cell complex maps were based on the macula cube scanning protocol, centered on the fovea that had a cube of 512 × 128 using an automated mGCC measurement and internal limiting membrane (ILM). The mGCC thickness measurements include the ganglion cell layer and inner plexiform layer (IPL). OCT scans with a signal strength >7 were included for analysis. Peripapillary RNFL thickness and mGCC measurements of PEX syndrome eyes and fellow eyes from each patient were assessed, and these values were compared.

### Optical coherence tomography angiography

OCT angiography was used to quantify the capillary-level vascular structures of the retina. All the scans were analyzed using OCT-A images that were automatically generated with the Cirrus OCT-A optical microangiography algorithm (Angioplex™ software, version 10.0, Cirrus; Zeiss, Dublin, USA). The software only reports superficial capillary plexus measurements that were calculated from the ILM to the posterior border of the IPL.

**Figure 1:**
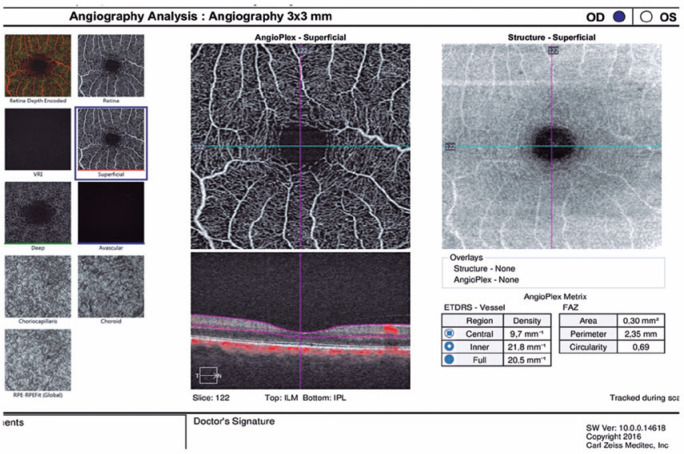
The optical coherence tomography angiography image is overlaid with an Early Treatment of Diabetic Retinopathy Study grid. The measurement tool (AngioPlex software, version 10.0; Carl Zeiss Meditec, Inc) provides vessel density measurements in the individual subfields (central, inner and full region) with 3 × 3-mm scan pattern.

Macular vessel density was calculated using the Early Treatment of Diabetic Retinopathy Study subfields software that quantified the vessel density of a local region of tissue. Measurements of the macular superficial vessel density were determined from 3 × 3-mm^2^ scans based on the fovea. Angioplex™ subdivides the scan into the following areas: a central region, an inner region, and a full region (Figure 1).

### Statistical analysis

The Shapiro-Wilk test was used to determine whether the data were distributed normally. Descriptive statistics included the mean and standard deviation for samples with normally distributed variables. BCVA assessments were converted into logarithm of the minimum angle of resolution (logMAR) for statistical analysis. Inter-eye asymmetry values of difference in peripapillary RNFL thickness, mGCC, and macular vessel density parameters were calculated as the absolute value of the difference between the values of the PEX syndrome eye and those of the fellow eye. P values <0.05 were considered to indicate statistical significance.

## RESULTS

Thirty patients with unilateral PEX syndrome, including 24 women and 6 men, were enrolled in the study. The mean patient age was 64.20 ± 7.05 (range, 58-77) y. There were no significant differences in the BCVA, IOP, central corneal thickness, cup-to-disc ratio, and the average mean deviation between the PEX syndrome eye and the fellow eye in the same subject (Table 1).

**Table 1:** Summary of the ophthalmic characteristics of the subjects

	PEX syndrome eye (n=30)	Fellow eye (n=30)	p∗ value
logMAR BCVA	0.30 ± 0.14	0.20 ± 0.10	0.105
IOP (mmHg)	14.8 ± 3.4	16.0 ± 1.9	0.236
CCT (µm)	554.8 ± 17.6	555.0 ± 9.3	0.969
Cup-to-disc ratio	0.62 ± 0.19	0.44 ± 0.12	0.139
Mean deviation (dB)	-0.6 ± 0.2	-0.5 ± 0.3	0.116

For normally distributed variables, results are shown as mean ± standard deviation.

Inter-eye asymmetry of the average peripapillary RNFL thickness and mGCC measurements were significant in the study group (p=0.03 and p=0.001, respectively). When peripapillary RNFL thicknesses were compared in different sectors, there was no significant inter-eye asymmetry except RNFL thickness in the inferotemporal, inferonasal, and superonasal sectors (Table 2).

**Table 2:** Fellow-eye Asymmetry of Peripapillary Retinal Nerve Fiber Layer Thickness, Macular Ganglion Cell Complex Measurements and Macular Superficial Vessel Density

	PEX Syndrome eye (n=30)	Fellow eye (n=30)	p^*^ value
RNFL thickness			
Average	79.2 ± 14.2	91.4 ± 16.3	0.038
Superotemporal	109.6 ± 20.3	123.2 ± 30.1	0.159
Superonasal	78.0 ± 8.3	94.8 ± 24.7	0.019
Nasal	66.6 ± 5.6	67.8 ± 13.9	0.760
Inferonasal	82.6 ± 27.7	105.6 ± 20.0	0.015
Inferotemporal	89.0 ± 31.0	124.0 ± 17.7	0.001
Temporal	59.8 ± 7.4	65.6 ±11.3	0.109
mGCC	54.6 ± 19.0	78.2 ± 16.5	0.001
Macular superficial vessel density			
Central region	8.2 ± 3.2	6.8 ± 2.8	0.536
Inner region	19.5 ± 2.3	17.3 ± 3.9	0.035
Full region	18.1 ± 2.4	16.2 ± 3.3	0.236

∗ Independent *t* test.

RNFL= retinal nerve fiber layer; mGCC= macular ganglion cell complex; PEX= pseudoexfoliation.

In macular superficial vessel density analysis, the average central, inner, and full region superficial vessel densities were 6.8 ± 2.8, 17.3 ± 3.9, and 16.2 ± 3.4 in PEX syndrome eyes and 8.2 ± 3.2, 19.5 ± 2.3, and 18.1 ± 2.4 in fellow eyes, respectively.

The average inner region superficial vessel density was significantly lower in eyes with PEX syndrome than in fellow eyes (p=0.035). However, there was no significant inter-eye asymmetry in the central and full region macular superficial vessel density between PEX syndrome eyes and fellow eyes (p>0.05), table 2.

∗ Independent *t* test. logMAR= logarithm of minimum angle of resolution; BCVA= best-corrected visual acuity; IOP= intraocular pressure; CCT= central corneal thickness; PEX= pseudoexfoliation.

## DISCUSSION

Pseudoexfoliation glaucoma is a secondary open-angle glaucoma that leads to more rapid visual field loss and may be more resistant to medical therapy^(14)^. It is essential to predict which patients with *PEX syndrome* are at higher risk of optic nerve damage because 5.3% of PEX syndrome patients may develop PEX glaucoma within 5 y and 15.4% may develop it within 10 y^(15)^. Early diagnosis is important because the visual field, which is considered as the gold standard for glaucoma diagnosis, remains unchanged until 25%-35% of the neurons are damaged. Structural tests, such as OCT, can enable the early detection of glaucomatous changes with cross-sectional imaging of the optic nerve head, RNFL, and macula^(16)^. In recent years, OCT-A, as a functional OCT technique, is widely used to identify vascular changes in the optic nerve head, peripapillary, and macula region in glaucoma.

Although the underlying pathologic mechanisms in PEX syndrome are not fully understood, the accumulation of PEX aggregates in the vessels of the anterior and posterior segments of the eye has been shown^(17)^. The Early Manifest Glaucoma Trial (EMGT) study demonstrated that PEX is a strong predictor of glaucoma progression independent of intraocular pressure and other risk factors^(18)^; research has suggested that diminished ocular blood flow may exacerbate glaucomatous damage^(19)^.

To our knowledge, no previous study has compared the inter-eye difference of retinal vessel density in subjects with unilateral PEX syndrome. In the present study, we found that peripapillary RNFL, mGCC, and superficial vessel density in the inner region of the macula were significantly diminished in PEX eyes as compared to that in fellow eyes.

In several studies, the peripapillary RNFL thickness is reportedly significantly thinner in eyes with PEX than that in healthy age-matched control eyes^(20)^. Yuksel and colleagues evaluate RNFL thickness in patients with unilateral PEX syndrome and compare these values with fellow eyes and age-matched healthy controls; they suggested that ocular blood flow disturbances owing to PEX material accumulation might cause inner retinal atrophy and thinner RNFL in eyes with pseudoexfoliation. In the fellow eyes, there was no significant difference in the RNFL measurements except for the temporal quadrant when compared with the controls^(21)^. Previous studies have shown that RNFL thickness has good diagnostic power for the detection of glaucoma. The GCC was also found to be an equally good predictor of optic nerve damage, and a good supplement for identifying patients with pre-perimetric glaucoma because GCC loss is significant in pre-perimetric glaucoma^(22,23)^. In our study, both, the average peripapillary RNFL thickness and the mGCC were thinner in PEX syndrome eyes than in fellow eyes of the same subject. The inter-eye asymmetry of these measurements was significant.

In recent publications, retrobulbar blood flow has been evaluated by measuring the choroidal thickness using spectral-domain OCT in patients with PEX syndrome, and useful information has been obtained. Goktas et al. showed that macular choroidal thickness was thinner in eyes with PEX syndrome than that in healthy controls, and a decrease in choroidal blood flow due to increased vascular resistance was suggested as a causal factor^(24)^. OCT-A studies reported vascular deterioration in eyes with suspected glaucoma^(25)^, with reduced macular vessel density in these eyes without measurable changes in mGCC^(26)^. Vascular dropout is considered an early sign of glaucomatous damage before detectable loss of visual field^(13)^. It is suggested that the decrease in vessel density at the early stage of glaucoma would cause earlier asymmetry between the eyes. Hou et al., evaluated the inter-eye asymmetry of vessel density and thickness values in glaucoma, glaucoma suspect, and healthy eyes; and found that optic nerve head and macular whole image inter-eye vessel density asymmetries were significantly greater in glaucoma suspects compared to healthy eyes. In contrast, mGCC and RNFL thickness asymmetry was not larger in glaucoma suspect compared to healthy eyes. This study demonstrates that inter-eye retinal vessel density asymmetry could be detected before significant retinal thickness asymmetry develops in glaucoma-suspect eyes^(27)^.

In the present study, we found significant inter-eye asymmetry in macular superficial vessel density in the inner perifoveal region of PEX eyes and fellow eyes. Chen et al., showed that the superficial layer vessel density decreased in primary open-angle glaucoma cases than that in healthy controls, and they demonstrated that whole vessel density had the highest diagnostic value, with perifoveal areas also showing significant changes^(28)^. Our results are consistent with those of OCT-A based studies, suggesting that vascular changes associated with glaucoma occur primarily in the superficial vascular complex of the retina^(29,30)^.

The wide variability in the structural features of the optic nerve head in a healthy population makes it difficult to detect early glaucomatous damage^(8)^. Anatomical symmetry between eyes is usually preserved in healthy eyes. Glaucoma is typically bilateral; however, it has asymmetric characteristics, especially in the early stages of the disease^(5)^. Hence, the inter-eye comparison may serve as a convenient reference for determining early-stage damage in glaucoma in comparison to controls with different genetic or environmental backgrounds.

There are certain limitations of our study. First, the small sample of participants and the retrospective nature of the study enabled us to demonstrate a causal relationship between PEX syndrome and glaucoma progression with the use of OCT-A. Second, the present study did not include peripapillary vessel density for comparison of PEX eyes and fellow eyes; this is an important parameter that must be considered. However, to our knowledge, this is the first study to provide evidence regarding inter-eye asymmetry of macular vessel density, peripapillary RNFL thickness, and mGCC in eyes with unilateral PEX syndrome. Thinner RNFL and mGCC and reduced macular superficial vessel density may be the factors that contribute to rapid progression to glaucoma in eyes with PEX syndrome.

In conclusion, there was significant inter-eye asymmetry in the inner region macular superficial vessel density, peripapillary RNFL thickness, and mGCC thickness in subjects with unilateral PEX syndrome in this study. Further studies are warranted to obtain a deeper understanding of the relationship between hemodynamic changes and disease progression in pseudoexfoliation.
